# A Case of Cæsarean Section for Obstruction of Labour by a Pelvic Tumour

**Published:** 1904-06

**Authors:** W. C. Swayne

**Affiliations:** Obstetric Physician to the Bristol Royal Infirmary; Professor of Midwifery, University College, Bristol


### A CASE OF CÆSAREAN SECTION FOR OBSTRUCTION OF LABOUR BY A PELVIC TUMOUR.

BY

W. C. Swayne, M.D., B.S.Lond.,

*Obstetric Physician to the Bristol Royal Infirmary; Professor of Midwifery, University College, Bristol.*

A. W., *primipara*, aged 22, was admitted to the Bristol Royal Infirmary on June 12th, 1903, on account of pains which she took to be labour pains. Her medical attendant on examination found a large tumour completely blocking the pelvis. She estimated that her pregnancy had advanced to the eighth month, but was uncertain as to its exact duration.

On examination of the abdomen it was found that the fundus of the uterus reached to the ensiform cartilage, the head of the fœtus was found to lie above the pelvic brim in the second vertex position, the fœtal heart was easily heard and strong movements felt. On vaginal examination a tense, elastic, globular tumour was felt blocking the pelvis to such an extent that two fingers could only pass it with great difficulty. In front the cervix could just be reached; it was soft and the os patulous. The tumour though elastic felt hard. No labour pains were present, but the usual intermittent uterine contractions could be made out. The pains ceased after the administration of an enema, which caused the evacuation of a number of scybalous masses.

As it was obvious that delivery *per vias naturales* was impossible, and as there was considerable doubt as to the real duration of pregnancy, it was decided to wait until labour came on and then deliver by Cæsarean section if necessary. The various alternatives were carefully considered. Vaginal puncture and delivery *per vias naturales* were rejected, since from the hardness of the tumour it seemed not unlikely that it might prove to be a fibroid, while if ovarian it was most probably a dermoid cyst. In the former case puncture would have been of no assistance, while in the latter, experience shows that vaginal puncture or incision in dermoids is often followed by prolonged suppuration, or even by sloughing of the tumour and the risk of infection and peritonitis, and, if adhesions should be present, the necessity for an abdominal section after delivery. The removal of the tumour by abdominal section and preliminary eventration of the pregnant uterus seemed undesirable. Premature labour would have certainly come on, a large incision would have been needed, owing to prematurity, the child's chance of surviving would be lessened, and since it was uncertain exactly how far pregnancy had advanced, the chances of the child's survival would be still further lessened should it prove that the duration of pregnancy was shorter than was supposed. Again, the presence of the head in the pelvic brim was likely to interfere with the extraction of the tumour if ovarian, while if it should prove to be a fibroid complete hysterectomy would be necessary. Even if by this method the removal of the tumour proved feasible, the superadded shock of labour which at this date in pregnancy appeared certain to come on after an abdominal section, and the amount of handling of the uterus that would be necessary, would prove a very serious added complication. It was therefore decided to wait until labour came on, and then to empty the uterus by Cæsarean section and deal with the tumour after this had been done. By this method only the emptied uterus would oppose the removal of the tumour if ovarian, while if fibroid the removal of the emptied uterus with the tumour would be a matter of far less difficulty.

Labour pains commenced on the evening of July 11th, and the membranes ruptured easily on the morning of July 12th.

*Operation.*—At 11.30 a.m. on July 12th the abdomen was opened by a seven-inch incision extending from an inch and a half above the umbilicus to an inch and a half above the symphysis pubis. A large ring pessary was then pressed firmly against the uterine wall and the uterus opened, the fœtus being seen lying within the membranes, which were opened after carefully packing sponges round the uterine incision, and the child extracted by the head. Two fingers were then hooked into the ends of the incision in the uterus and the latter pulled out of the abdominal wound. The edge of the placenta was seen to present at the wound; it was removed, and the uterus enveloped in towels wrung out of hot sterilised saline solution, kept firmly compressed by Dr. Nixon. It retracted well, and there was very little bleeding. The tumour was then removed with some difficulty owing to adhesions, its pedicle ligatured, and the end of the pedicle oversewn with fine silk so as to bury the raw surface of the stump in peritoneum. The uterine wound was then sewn up with four layers of suture—first a row of interrupted medium silk, then two layers of continuous catgut uniting the muscle but avoiding the mucous membrane, and, finally, a continuous peritoneal suture of fine silk burying the whole of the foregoing. (The interrupted silk sutures included the whole uterine wall and peritoneum, omitting the mucous membrane, and were used to reinforce the catgut sutures.) The peritoneal cavity was now flushed out with a couple of washing jugs full of hot sterile saline solution, and as the oozing from the adhesions had ceased the abdomen was closed by a continuous silk suture of the peritoneum and interrupted silkworm-gut sutures taking up skin, muscle and fascia. Previous to closing the abdominal wound the uterus was firmly compressed and a certain amount of blood expelled from the vagina. A hypodermic injection of ergotin had been previously given. During the operation, as the patient showed signs of shock, Dr. Stack performed saline transfusion.

In such an operation as this much depends on the ability of the assistants, two of whom are necessary in addition to the anæsthetist. To one of the two should be committed the care of the uterus, and his entire duty should be to keep up firm manual compression, the organ being enveloped in hot sterilised towels so long as it is outside the abdominal cavity; this can be done without interfering with the operation.

The success of the operation in this case was largely due to the able assistance rendered to the operator by Mr. N. A. W. Conolly (resident obstetric officer), to whom I am indebted for the notes of the case, and Dr. Nixon, who commanded the uterus so successfully that the total amount of blood lost was not more than one usually sees in a normal confinement.

The patient suffered very little from shock after the operation. She had a slight attack of hemorrhage from the vagina in the afternoon, but it was not sufficient to cause anxiety. Her further progress was uneventful until the seventh day, when some fœtor of the vaginal discharge was noticed and the uterus washed out. The sutures were removed on the tenth day, and on the thirteenth a stitch abscess developed, from the sinus of which the silk peritoneal suture was removed. Her further progress was unimpeded. The child, a well-developed male, thrived surprisingly (at date of writing he is 6½ months old).

I examined the patient a few weeks ago. The uterus was then movable, of normal size and in normal position. A little thickening could be felt about the ovarian stump.

The tumour was a dermoid cyst of the right ovary, containing in one part hair and the other constituents of a dermoid cyst, while the remainder seemed to be composed of the ordinary constituents of a multilocular cyst.

---

